# Supplementary material for: Anodal M1 tDCS enhances online learning of rhythmic timing videogame skill
Source: PLoS One. 2024 Jun 13;19(6):e0295373. doi: 10.1371/journal.pone.0295373 (PMC11175489; doi:10.1371/journal.pone.0295373)
Supplement: Attachment — Submitted filename: plosone02024.pdf [file pone.0295373.s001.pdf]

I have thoroughly reviewed the manuscript titled "Anodal M1 tDCS enhances online learning of rhythmic timing videogame skill" and found the study to be a valuable contribution to our understanding of motor learning, particularly within the context of complex real-world tasks. The manuscript is well-written and provides significant insights into the potential of M1 anodal transcranial direct current stimulation (a-tDCS) to enhance motor learning, especially in the domain of complex real-world tasks, such as the Step Mania video game. However, to strengthen the manuscript, I recommend considering the following points for incorporation.

Introduction:

I suggest refining the introduction to lead the reader more effectively to understand the formulation of the research question.

For instance, the authors referenced lab-based tasks on page 4, yet I missed a discussion on the other studies conducted with more complex tasks to offer additional context, since they have only mentioned an increasing number of tasks expanding complexity but not explored these studies.

It would also be valid to ensure clarity in articulating the specific study's objectives and specify hypotheses related to anticipated changes in skill learning resulting from M1 a-tDCS application.

Finally, focus the introduction more explicitly on the study's objective. For instance, information about multiple sections (page 3, line 1-2) may be considered less pertinent than the other points I mentioned and could be streamline for better alignment with the study's main focus.

Methods:

The method is well-written, with detailed and easily-followed methodology. I appreciate the care taken in creating a score and reporting it in such a meticulous manner. However, I would like to offer some suggestions:

Please include information on whether a sample size calculation was conducted.

Could the authors provide clarification on whether the study was retrospective? Additionally, it would be valuable to know if the data analyzed in this study has also been considered in another publication.

It would be appreciated if the authors could provide details on the randomization procedure and specify if participation was voluntary. Consider following guidelines for reporting your experiment.

Could the authors enhance the resolution of Figure 1?

An explanation of the rationale behind choosing current intensity and stimulation duration would be helpful.

Could the authors provide clarification on whether participants' naivety to rhythm games served as an control variable in the study? If not, I think it would be beneficial to include a comment on this aspect in the discussions. However, in the discussion (pag 23), it is mentioned that participants were naive to rhythm games – was this an exclusion criterion then? If so, it would be helpful to add this information to the description of exclusion criteria.

Results:

Consider incorporating the results of the post-test into Figure 2 to provide an overarching view of the experiment and the longevity of effects post-training.

Discussion:

The discussion section of the paper addresses various crucial points regarding the outcomes derived from the application of M1 a-tDCS in acquiring motor skills within a timing-based video game. Here are some suggestions to further elaborate on the discussion:

Elaborate on the possibility that the a-tDCS group started with more errors in baseline in Figure 4C.

Discuss additional limitations and suggest areas for future research.

Given that the study's sample comprises a healthy population, consider rephrasing the last sentence of the discussion (page 24, l. 14-16) to emphasize the need for additional research within a rehabilitation context.

Minor comments:

Italicize the *p*-values

Spell out PM and SMA before using the acronyms (pag 22)
